# Comparative Analysis of Four Different Intraoral Scanners: An In Vitro Study

**DOI:** 10.3390/diagnostics14131453

**Published:** 2024-07-08

**Authors:** Lucian Toma Ciocan, Vlad Gabriel Vasilescu, Sabina-Ana Răuță, Mihaela Pantea, Silviu-Mirel Pițuru, Marina Imre

**Affiliations:** 1 Discipline of Dental Prosthetics Technology, Faculty of Dentistry, “Carol Davila” University of Medicine and Pharmacy, Dionisie Lupu Street, No. 37, District 2, 020021 Bucharest, Romania; lucian.ciocan@umfcd.ro; 2 Faculty of Dentistry, “Carol Davila” University of Medicine and Pharmacy, Dionisie Lupu Street, No. 37, District 2, 020021 Bucharest, Romania; sabina.rauta@stud.umfcd.ro; 3 Discipline of Prosthodontics, Faculty of Dentistry, “Carol Davila” University of Medicine and Pharmacy, Dionisie Lupu Street, No. 37, District 2, 020021 Bucharest, Romania; marina.imre@umfcd.ro; 4 Discipline of Organization, Professional Legislation and Dental Office Management, “Carol Davila” University of Medicine and Pharmacy, Dionisie Lupu Street, No. 37, District 2, 020021 Bucharest, Romania; silviu.pituru@umfcd.ro

**Keywords:** prosthodontics, digital impression, accuracy, intraoral scanner, ISO assessment, dental arch

## Abstract

(1) Background: Intraoral scanners undergo rapid advancements in hardware and software, prompting frequent updates by manufacturers. (2) Aim: This study aimed to quantitatively assess the precision of full dental arch digital impressions obtained from four different intraoral scanners: Trios 5—3SHAPE, Copenhagen, Denmark, CEREC Primescan— Dentsply Sirona, New York, NY, USA, Planmeca Emerald S—Planmeca Oy, Helsinki, Finland, and Medit i700—Medit Corp, Seoul, Republic of Korea. (3) Methods: A maxillary virtual dental model (digital master model) was created in accordance with ISO standard 20896-1. Subsequently, a 3D-printed model was obtained from the master model’s STL file and scanned 15 times consecutively with each scanner. STL files were aligned with the master model’s STL using Medit Link—Medit Design software v.3.1.0. The accuracy was evaluated by measuring deviations in micrometers between each scanner’s scans and the master model. (4) Results: The study revealed variations in accuracy ranging from 23 to 32 µm across scans of the same dental arch, irrespective of the scanner used and scanning strategy employed. The anterior regions exhibited higher precision (Mean Absolute Deviation of 112 µm) compared to the posterior regions (Mean Absolute Deviation of 127 µm). Trios 5 demonstrated the smallest deviation (average 112 µm), indicating superior accuracy among the scanners tested. Emerald S and Medit i700 exhibited balanced performance (average 117 µm and 114 µm, respectively), while Primescan consistently displayed high deviation (average 127 µm). (5) Conclusions: Based on clinically accepted thresholds for accuracy in intraoral scanning, which are typically 200 µm for full arch scans, Trios 5 surpasses these benchmarks with its average deviation falling within the 200 µm range. Emerald S and Medit i700 also meet these standards, while Primescan, although showing high overall deviation, approaches the upper limit of clinical acceptability. Considering the limitations of an in vitro investigation, the findings demonstrate that each intraoral scanner under evaluation is capable of reliably and consistently capturing a full arch scan for dentate patients.

## 1. Introduction

Intraoral scanning devices have undergone rapid technological advancements, characterized by continual updates in both hardware and software capabilities from manufacturers. These advancements have significantly expanded their applications across various dental specialties, including orthodontics, restorative dentistry, oral surgery, prosthodontics, and dental technology. In contemporary digital dentistry, the precision improvements and specific technological differences in intraoral scanners play a crucial role. These scanners generate digital data files essential for initial diagnosis, treatment planning, and treatment execution, highlighting the critical need for highly accurate 3D data collection.

Despite the proliferation of intraoral scanning technologies, dental professionals face challenges in selecting the most appropriate scanner for their specific clinical needs. Factors such as market distribution, hardware design, and cost often influence these decisions. Moreover, the scientific literature currently lacks updated information on the comparative accuracy and performance of different intraoral scanners, hindering dental practitioners from making well-informed decisions regarding scanner selection tailored to diverse clinical contexts [1,2].

Recent developments have seen the emergence of open-source software solutions designed to facilitate comprehensive comparative analyses of intraoral scanners. These tools, such as those discussed in recent studies (e.g., Autodesk Meshmixer v.3.5.4, Cloud Compare v.2.12), provide standardized frameworks for evaluating scanner accuracy and performance across various dental applications [3]. However, there remains a need for empirical data that systematically evaluates and compares the precision and clinical efficacy of new-generation scanners in real-world clinical settings.

Recognizing this gap, the Institute of Digital Dentistry emphasizes the necessity for a rigorous comparative analysis to evaluate the accuracy and clinical efficacy of new-generation scanners. Therefore, this study aims to fill this gap by systematically evaluating and comparing the accuracy of four leading intraoral scanners for full arch dentate digital impressions. By providing empirical data on the performance of these scanners, we aim to offer valuable insights that can guide dental professionals in selecting the most suitable technology for various dental applications.

### 1.1. Null Hypotheses

The study’s first null hypothesis posits that the accuracy of full arch impressions captured by intraoral scanners is equivalent to that of modern conventional impression materials. This hypothesis aims to address the fundamental question of whether digital impression technology can achieve comparable or superior accuracy compared to traditional impression materials. Traditional materials, such as polyvinyl siloxane (PVS) and alginate, have been used for decades and are considered standard for dental impressions. Assessing if intraoral scanners can match or surpass this established accuracy is crucial for determining their efficacy and suitability in clinical practice. By testing this hypothesis, the study focuses on providing evidence on the accuracy capabilities of intraoral scanners relative to conventional methods.

The second null hypothesis suggests that the accuracy of various updated intraoral scanners is comparable. This hypothesis aims to explore whether advancements in scanner technology have led to significant differences in accuracy among different models, or if they yield similar outcomes in digital impression accuracy. As manufacturers continually update their scanner models with improved hardware and software, understanding the comparative accuracy of these advancements is essential for dental practitioners and researchers. By testing this hypothesis, the study seeks to provide insights into the consistency and reliability of different intraoral scanners in capturing digital impressions.

By testing these null hypotheses, the study aims to fill the existing gaps in understanding the comparative accuracy of intraoral scanners relative to traditional methods and among different scanner models. This approach not only supports the scientific rigor of our research but also provides actionable insights for dental professionals navigating the landscape of digital dentistry.

### 1.2. Aim

The primary objective of our research was to undertake a meticulous comparison of the accuracy exhibited by four distinct intraoral scanners: Trios 5—3SHAPE, Copenhagen, Denmark, CEREC Primescan—Dentsply Sirona, New York, NY, USA, Planmeca Emerald S—Planmeca Oy, Helsinki, Finland and Medit i700—Medit Corp, Seoul, Republic of Korea.

In our quest for comprehensive evaluation, we meticulously scrutinized the performance metrics of each scanner, examining their capabilities in capturing detailed digital impressions of full arch dentate structures. By conducting a head-to-head comparison of these intraoral scanners, we aimed to elucidate their respective strengths, weaknesses, and overall suitability for diverse clinical applications. Through the measurement and analysis of the digital impressions generated by these scanners, we sought to provide valuable insights into their precision, accuracy, and reproducibility.

Furthermore, by including scanners from different manufacturers and geographical locations, we aimed to offer a comprehensive assessment that encompasses a diverse range of technological advancements and design philosophies. This approach allowed us to explore potential variations in performance attributable to differences in engineering, software algorithms, and manufacturing standards.

Ultimately, our study endeavors to equip dental professionals with evidence-based guidance and data to inform their decision-making processes when selecting an intraoral scanner for clinical practice. By shedding light on the comparative accuracy of these leading intraoral scanners, we aim to empower clinicians with the knowledge needed to make informed choices that optimize patient care and treatment outcomes.

## 2. Materials and Methods

Each scanner was subjected to rigorous testing protocols and standardized procedures to ensure consistency and reliability in our comparative analysis.

In our study, we employed Blender software (Blender v.3.3 LTS, the Blender Foundation, Amsterdam, The Netherlands), a versatile and widely utilized digital modeling tool [2,4], to meticulously construct a digital test object. This digital model was meticulously designed to replicate the geometry and specifications outlined in ISO standard 20896-1, titled “Dentistry-Digital impression devices Part 1: Methods for assessing accuracy” [5]. The aim was to ensure alignment with the internationally recognized standards for evaluating the accuracy of digital impression devices. This standard specifies the requirements for digital dental models, ensuring geometric accuracy and dimensional stability.

Following the guidelines set forth by the ISO standard, the digital test object featured a gauge ball with a diameter of 6 mm. Adhering closely to these specifications was imperative to maintain consistency and comparability with the established testing protocols [5]. The utilization of Blender software facilitated precise control over the design and dimensions of the digital model, enabling us to create a faithful representation of the standardized test object.

**Figure 1:**
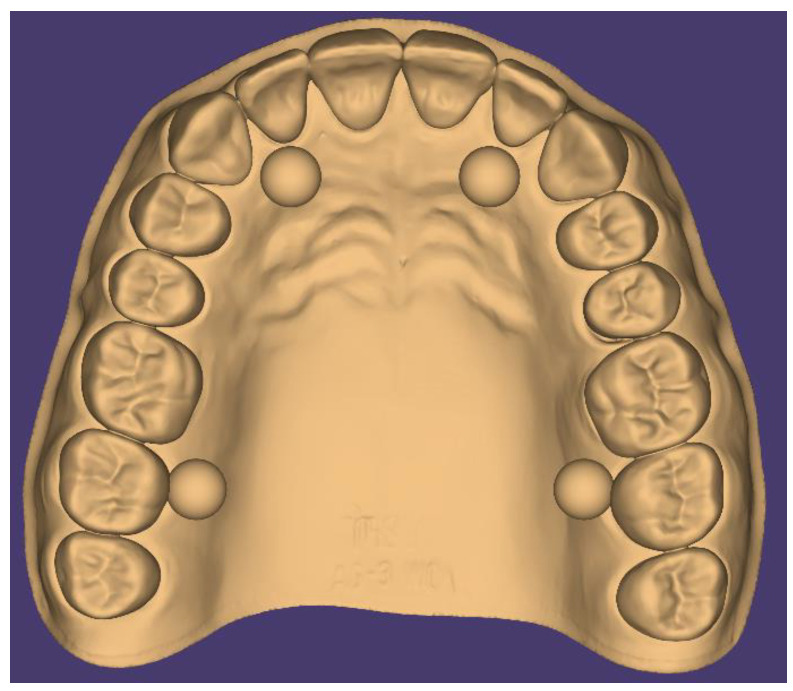
Digital test object created in Blender according ISO standard 20896-1.

Figure 1 depicts the digital representation of the test object, showcasing its geometry and dimensions. Through attention to detail and adherence to ISO guidelines, we aimed to create a standardized testing environment that would facilitate an accurate and reliable assessment of the performance of the intraoral scanners under evaluation.

Table 1 provides a comprehensive overview of the specifications and parameters governing the construction of the digital test object. By documenting these details, we sought to ensure transparency and reproducibility in our experimental methodology.

Overall, the construction of the digital test object in accordance with ISO standards underscores our commitment to rigor and precision in experimental design. This standardized approach lays the foundation for objective and meaningful comparisons of the accuracy exhibited by the evaluated intraoral scanners, contributing to the advancement of knowledge in the field of digital dentistry.

The dimensions under scrutiny for the full dental arch encompass the six distances delineated between the centers of the gauge balls, spanning from point “a” to “b”, “b” to “c”, “c” to “d”, “d” to “a”, and the two diagonals extending from the center of ball “d” to ball “b” and from the center of ball “a” to ball “c”. This measurement approach facilitates comprehensive insights into both the sagittal and transverse dimensions of interest.

Each of these six distances, denoted as D a-b, D b-c, D c-d, D a-d, D a-c, and D b-d, will be measured and documented, serving as reference or true values.

In our experimental setup, we utilized Planmeca Creo C5 3D printer by Planmeca Oy, Helsinki, Finland to seamlessly import and translate the digital design of the test object into physical form [6]. This 3D printer, renowned for its precision and reliability, played a pivotal role in the fabrication process, ensuring the accurate reproduction of the digital model with fidelity.

**Figure 2:**
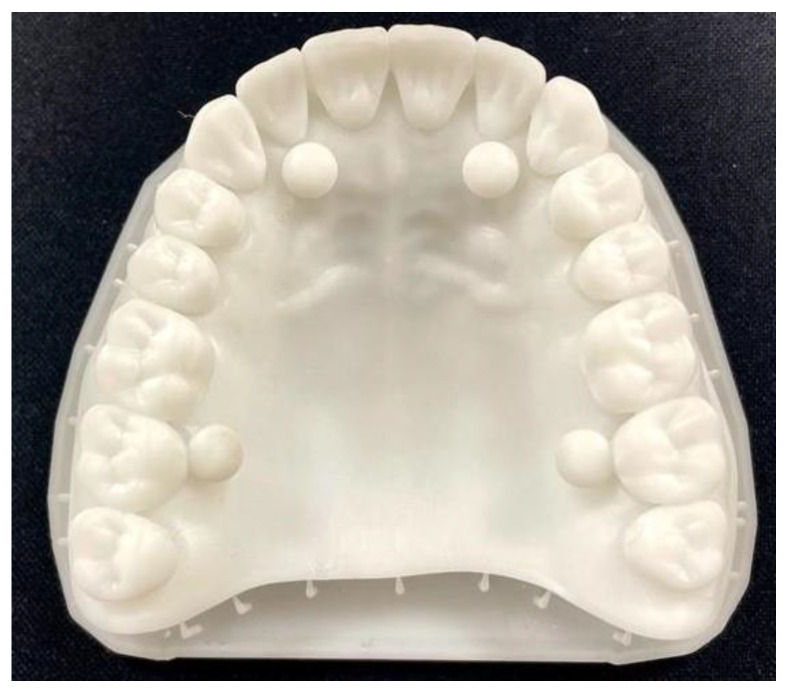
The test object obtained by 3D printing.

Figure 2 illustrates the Planmeca Creo C5 3D printer in action, showcasing its advanced capabilities in transforming digital designs into tangible objects with remarkable accuracy and detail. The printer’s technology and innovative features enabled it to execute the printing process with exceptional precision, resulting in high-fidelity replicas of the digital test object.

By leveraging the Planmeca Creo C5 3D printer, we were able to produce physical replicas of the digital test object with the utmost precision and consistency. This facilitated the creation of standardized test specimens that closely adhered to the specifications outlined in ISO standard 20896-1 [5], ensuring alignment with internationally recognized testing protocols. For the validation of the printed model, the following equipment was used: Nikon XTH225 ST Reflection target by Nikon Co., Tokyo, Japan, and the analysis software was VGStudio MAX v.2023.1. The steps were as follows: The sample was positioned on the tray inside the equipment. Parametrization, a scanning process, and model reconstruction were carried out using the CT Pro 3D program from Nikon. The model was then aligned and imported into the VGStudio MAX 2023.1 software, where the brightness and contrast were adjusted to achieve the best image quality, followed by taking measurements. For confirmation, the Geometry Elements module and Measurement module were used, and the program can accordingly indicate whether our dimensions are accurate.

In our study protocol, attention was paid to ensuring the scanning procedures were standardized and consistent across all intraoral scanners evaluated. Each test object underwent 15 scans using the respective intraoral scanner models, including Trios 5—3SHAPE (DK), CEREC Primescan—Dentsply Sirona (US), Planmeca Emerald S—Planmeca Oy (FI) and Medit i700—Medit Corp (KR). To minimize potential sources of bias, operators adhered to uniform scanning distances and movement protocols throughout the scanning process [7,8]. The scanning strategy started at the molar occlusal surface. The scanner was subjected to a sweeping motion (wiggling motion) when passing the centrals, continuing until reaching the last molar. Then, the buccal side, the palatal side, and the palate were scanned. All scans were performed on a phantom head. To minimize operator variability, all scanning procedures adhered strictly to a standardized protocol for all 15 consecutive scans executed with each scanner. This approach ensured comprehensive coverage of the dental arch and consistent scanning sequences across all scanners.

Moreover, environmental factors were rigorously controlled to maintain uniformity across scanning sessions. Scans were conducted in identical environments with consistent levels of humidity, temperature, and illumination, thereby minimizing potential variations that could affect scan quality and accuracy [5,9]:

> Temperature: maintained at 23 ± 1 °C, monitored with a digital thermometer calibrated per ISO [5].
> Humidity: kept constant at 40 ± 5%, monitored using a hygrometer.
> Lighting: controlled at 15,000 lumens to ensure consistent illumination across scanning environments, in a room with a surface of about 90 m^2^.
> Master Model Handling: the master model had a fixed position on a phantom head to prevent physical alterations or deviations during scanning sessions.

This stringent approach ensured that any observed differences in scan accuracy could be attributed to the inherent characteristics of the scanners rather than external environmental influences [10].

For the analysis of scanned data, a standardized software program, Medit Link—Medit Design v.3.1.0, was chosen for its capabilities in aligning and analyzing digital scans [11]. This software facilitated the precise alignment of scanned data with the reference master model using the following superimposition strategy:

Superimposition strategy: The ‘Alignment Mode tool’ in Medit Link—Medit Design software allowed for the independent alignment of each scan with the reference model. The ‘Align target data separately’ option was utilized to optimize alignment accuracy, supplemented by automatic alignment features to minimize manual errors. This approach ensured the reliable comparison and measurement of deviations between scanned objects and the master model. The deviation analysis involved quantifying discrepancies between the reference master model and scanned objects. Medit Link—Medit Design software’s ‘Deviation Display Mode’ provided detailed deviation maps and tabular reports, presenting error margins and deviations in micrometers (µm). The calibration of measurement tools and software parameters was performed regularly to maintain accuracy and reliability throughout the analysis process.

**Figure 3:**
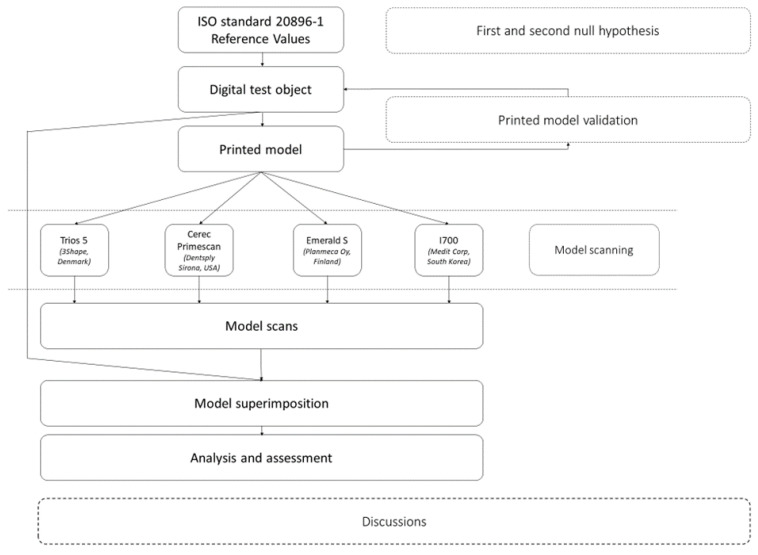
Method flow scheme.

It is worth noting that every scan conducted using the four evaluated scanners strictly adhered to this standardized protocol, and the scan setup used the study model, ensuring consistency and reliability in the assessment of scan accuracy across different scanner models. By employing a rigorous and systematic approach to data acquisition and analysis, we aimed to provide robust and reliable insights into the comparative performance of intraoral scanners in digital impression accuracy (Figure 3).

## 3. Results

**Figure 4:**
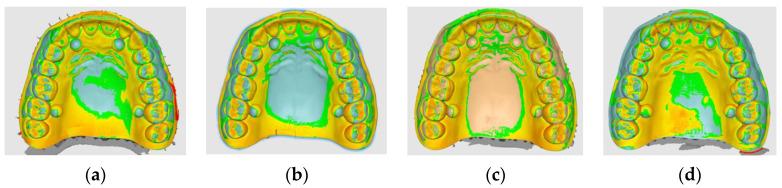
Overlapped data (15 scans each) for the four analyzed scanners. (**a**) Trios 5; (**b**) CEREC Primescan; (**c**) Emerald S; (**d**) i700.

Figure 4 shows the appearance of the overlapped data for the four distinct scanners, and Table 2 lists the associated values.

The key statistical measures used in this study are described below [12]:

Mean and Median: These measures provide the central tendency of the data. The mean is the average value of all measurements, while the median is the middle value when the data are ordered from least to greatest. These metrics help in understanding the typical deviation value for each scanner.

Mean Absolute Deviation (MAD): This metric measures the average absolute deviation of each value from the mean, providing insight into the consistency of the deviations. A lower MAD indicates higher precision.

Root Mean Square Error (RMSE): RMSE is the square root of the average of the squared differences between each scan value and the mean. It gives a quadratic measure of deviation, emphasizing larger errors more than smaller ones. RMSE is particularly useful in understanding the overall accuracy of the scans.

Standard Deviation (SD): SD measures the dispersion of the deviation values around the mean. It indicates how spread out the values are. A lower SD signifies more consistent accuracy.

Variance: Variance is the square of the standard deviation, providing a measure of how much the values differ from the mean. It is used to understand the degree of variation within the dataset.

Trueness (90–10)/2: Trueness is calculated as the difference between the 90th and 10th percentile deviation values divided by two. This metric indicates the accuracy of the measurements by showing the spread of the middle 80% of the data.

Tolerance: Tolerance represents the percentage of deviations within a predefined acceptable range, providing a measure of how often the scanner’s accuracy falls within clinically acceptable limits.

The results show that there are variations between scans for the same dental arch, regardless of the scanner, even if the same protocol and scanning technique were followed. Compared to the posterior area, the anterior area produced more precise results.

In comparison to the control sample, every assessed scanning system had both positive and negative deformations. The Emerald S system is the most likely to generate a primarily positive deformation out of all the systems that have been studied. According to this perspective, the Trios 5 system has the smallest deformation band, while the Emerald and i700 systems are more balanced. The i700 system’s scanning accuracy has a more consistent average scanning rate (average of 6 microns). The Trios 5 scanning system has the highest absolute accuracy (absolute average of 0.112 mm) among the evaluated systems.

**Figure 5:**
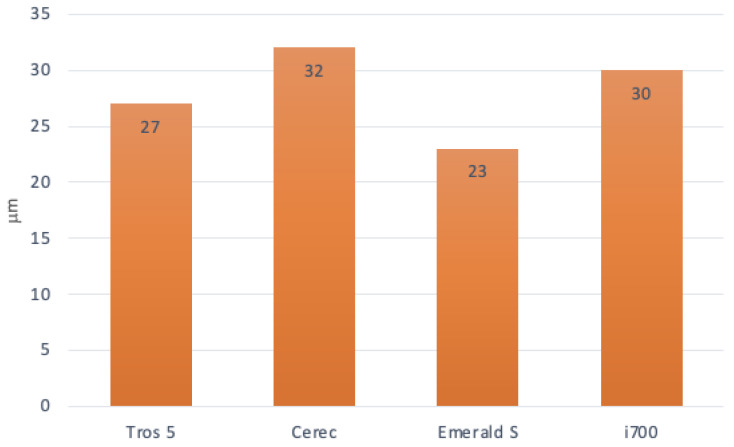
Average difference between two scans with the same system of the test sample.

The results for every sample show that there are variations between 23 and 32 μm between scans for the same dental arch, regardless of the scanner (hardware with particular software associated with it) (Table 2). Figure 5 illustrates that the maximum variation in standard deviation values across all test scans was 32 μm).

**Figure 6:**
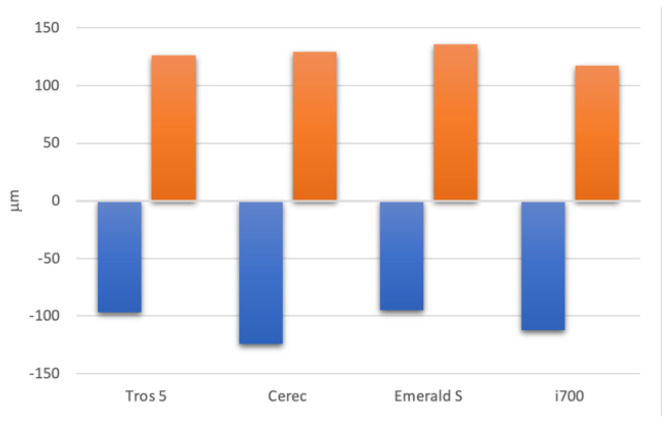
The average scanning deformations of the test object of the analyzed systems.

**Figure 7:**
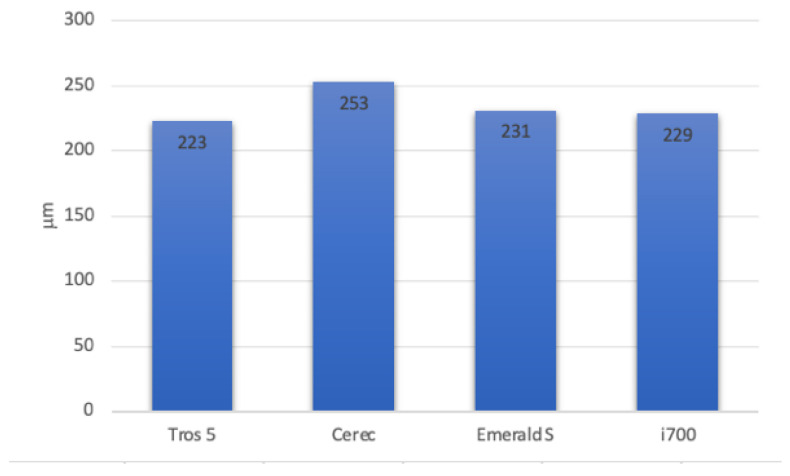
Variation in deformations between the minimum and maximum limits of different scanning systems (deformation band).

After analyzing the data, it was found that, in comparison to the control sample, every assessed scanning system had both positive and negative deformations. (Figure 6). The Emerald S system is the most likely to generate a primarily positive deformation out of all the systems that have been studied. According to this perspective, the Trios 5 system has the smallest deformation band, while the Emerald and i700 systems are more balanced (Figure 7).

The variance and standard deviation values, which serve as indicators of the scan accuracy, were discovered to vary even though the same scanning method and operator were used to record the results of the various scanning assemblies that were evaluated.

**Figure 8:**
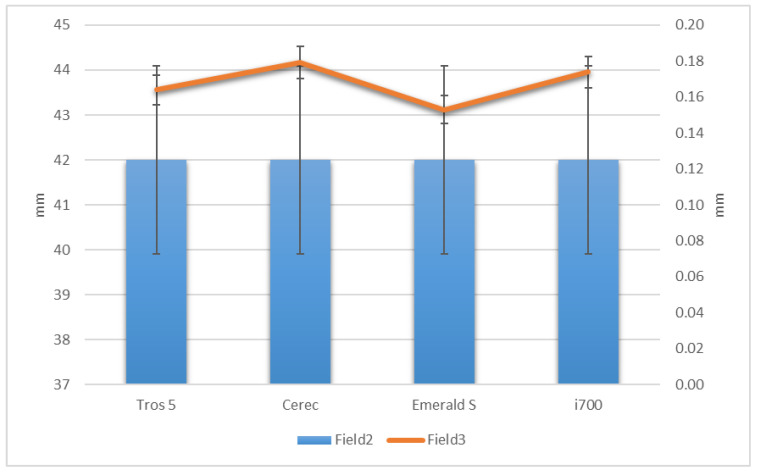
The standard deviation of the digital impressions made with the analyzed systems.

For example, Table 2 shows that, in comparison to the other three evaluated systems, the Emerald S system had the lowest variation value (0.023 mm). Additionally, as seen in Figure 8, the Emerald S system generated the lowest standard deviations (0.153 mm) when compared to the Trios 5 (SD = 0.164 mm), i700 (0.173 mm), and CEREC (0.179 mm) systems. This demonstrates that when compared to the other systems examined, the Emerald S system has a higher accuracy than the other systems tested.

## 4. Discussion

Accuracy is defined in ISO 5725-1 as the combination of trueness and precision [13].

In digital dentistry, trueness and precision can be evaluated in an incredible variety of ways, frequently not in compliance with metrological and ISO standards. When determining accuracy and truthfulness, there are some rules to follow. A comprehensive framework for the evaluation processes is provided by several ISO standards in the case of linear (one-dimensional) distance measurements. This kind of framework is used in current research. Currently, no ISO standard applies to surface comparison methods (multidimensional measurements). An evaluation procedure that follows the basic criteria of trueness and accuracy is suggested for that instance, and it expands the ideas for one-dimensional cases to multidimensional measures [14].

The method of evaluation of accuracy, precision, and trueness (bias) for single-distance measurements or single dimensions of interest (one-dimensional measures) can be entirely based on ISO standards [5,13,14]. The evaluation process of trueness and precision in surface comparison methods (multidimensional measures) can be extended from the ideas for one-dimensional situations through the use of the qualitative definitions of trueness and precision. When using surface comparison methods, it is necessary to specify a meaningful quantitative measure (such as RMSE, quantiles, absolute mean, or other values) that accurately describes the distance distribution. The presence or absence of a normal distribution for the distance values should serve as the basis for the quantitative measurement [14].

A significant feature of this study is that it adhered strictly to the ISO criteria for evaluating the accuracy of digital impression devices. Since non-standardized models are used as samples in other comparable studies [14], the distance and subsequent measure may differ.

According to ISO standard 20896-1, Dentistry—Digital impression devices Part 1: Methods for assessing accuracy [5]: a digital impression device is a handheld scanner designed for the oral cavity combined with computer hardware and software that generates a three-dimensional numerical description of the scanned surfaces. A dimension of interest is the distance between test object features that must be independently measured as a true value or reference, and the digital impression device must estimate this distance using a predetermined scanning technique. The hand-held scanning device is a camera or similar sensor which may be moved freely to catch the light that is reflected or diffusely scattered from a surface, turning the data into a series of numbers that can be used to calculate ranges and related directions to the surface. The same software was used to evaluate all the scan data.

Conventional workflows assume the use of conventional impression materials. Elastomeric materials, which experience a certain type of shrinkage due to the chemical polymerization reaction, are the conventional impression materials used for precise impressions in dental prosthesis [15]. The ISO 4823:2021 standard allows a dimensional change of less than 1.5% [16], while the ANSI/ADA specification no. 19-2022 specifies a maximum negative change in dimensions should be 0.5% after a minimum of 24 h, regarding the polymerization shrinkage of elastomeric impression materials [17]. According to the specialized literature and manufacturer brochures, the average polymerization shrinkage of elastomeric impression materials is approximately 0.4–0.6% for condensation silicones and 0.15–0.2% for addition silicones and polyethers [18,19,20]. These values vary depending on the preparation method, impression technique, and consistency, but they tend to be within the previously mentioned ranges [18]. According to an analysis of this data for the test sample’s dimensions based on the longest distances between landmarks (42 mm) (see Table 1), the condensation elastomers would have a polymerization shrinkage at the impression level of the test sample of 0.4–0.6% × 42 mm = 168–252 μm and the addition silicones and polyethers—conventional materials thought to be the most reliable—would have a shrinkage varying between 0.15–0.2% × 42 mm = 63–84 μm for the test samples. For the dimensions of the test object ANSI/ADA19:2022, the specification allows a polymerization shrinkage of 0.5% × 42 mm = 210 μm, while the ISO 4823:2021 standard allows a polymerization shrinkage of 1.5% × 42 mm = 0.6 mm (see Figure 4). In this regard, the digital impression systems (Trios 5 by 3SHAPE, CEREC Primescan by Dentsply Sirona, Emerald S by Planmeca, and i700 by Medit) all meet the requirements for the negative deformation value (contraction equivalent) that conventional impression materials must meet; the average negative deformation of these systems is lower, but comparatively slightly greater than that of contemporary addition silicone elastomers and polyethers [14,19,20,21,22] at the standard dimensions of dental arches (see Figure 4). There are studies that show a reduced deformation of digital systems compared to conventional impressions made with additive silicones, but through a two-time impression technique [23]. In conclusion, modern scanning technologies do not produce clinically noticeable relative deformations. This is contradictory to some results from previous assessments, which showed that traditional full-arch polyvinylsiloxane impressions were more accurate than two direct optical scanners, with every deviation being less than 100 μm [24].

**Figure 9:**
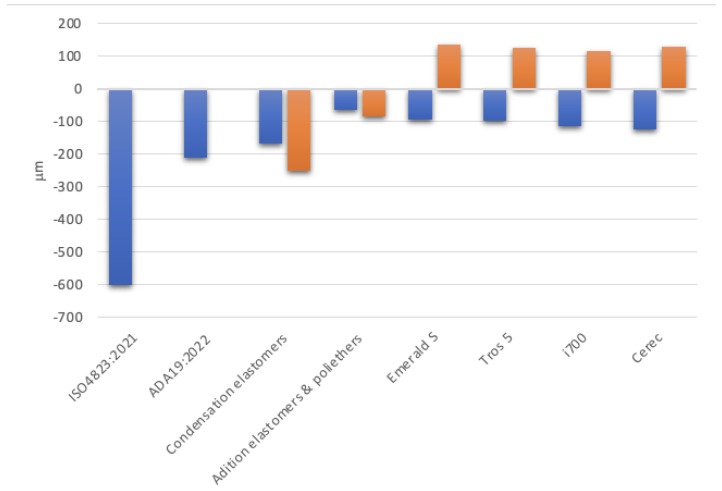
Accuracy of analyzed digital impression systems related to conventional elastomeric materials and their ISO and ADA standards.

In this study, the first null hypothesis was rejected, indicating significant variations in full arch impression accuracy between digital intra-oral scanners and conventional impression materials (Figure 9).

From this perspective, our findings match up with the specialized literature and demonstrate that all digital scanning systems under analysis have precision levels significantly higher than the standards required for conventional impression materials for dental arch impressions, indicating a similar degree of trueness between digital and conventional systems [25]. Moreover, our results support the assertion that validated digital impression systems produce clinically acceptable outcomes, positioning them as reliable alternatives to traditional impressions. Studies have demonstrated that the trueness of intraoral scanners (such as CEREC Omnicam and Trios 3) and conventional addition silicone and polyether were comparable. Higher local deviations throughout the entire arch can be displayed using digital impression devices. Compared to the posterior area, the anterior area produced more precise results [26,27]. Depending on the clinical indications, the digital impression may be used as a substitute of the conventional impression [28].

Due to variations in full arch impression accuracy observed across all digital intra-oral scanners under evaluation (see Table 2), the second null hypothesis was rejected. These discrepancies highlight the need for tailored scanning protocols and the careful consideration of scanner-specific performance characteristics in clinical practice.

It can be observed that the Emerald S acquisition system is the most stable in multiple scans when compared to the other systems studied, even though the maximum difference between the standard deviations of the four systems under analysis is only 26 microns. This finding has no considerable clinical significance. These findings are consistent with other recent studies that show that digital technology is less technique sensitive than traditional impression taking and that full arch impression accuracy improves with generational changes [29,30,31,32,33].

This study’s adherence to the ISO standard for evaluating the accuracy of digital impression devices is a significant feature. Different studies have considered that various standardized model types will be scanned by multiple technologies. Their outcomes are comparable, even though the ISO standard was not followed [1,24,25]. For all scanners, accuracy decreases as the scan distance is increased. Trueness varied in the scanner research, although the accuracy was often identical. For every scanner, the accuracy of diagonal scanning decreased. Therefore, the dentist has to choose a good scan pattern and use extra caution when scanning the entire arch. When compared to other scanners used in various studies, the Trios series displayed the best scan findings [1].

A recent meta-analysis [1] that examined 26 distinct scanners concluded that, despite some differences across the systems, IOS systems are precise enough to generate digital impressions of the entire arch that are suitable for clinical use. Under different clinical circumstances, the accuracy of IOSs and new generation scanners for entire arches can vary; however, there is an obvious tendency for accuracy increasing with time as IOS technology progresses [1,34,35].

In our study, among the examined scanners, the i700 system’s scanning accuracy has a more consistent average scanning rate (average of 6 microns). Based on our findings, it appears that the Medit i700 system represents an important improvement over the earlier i500 system, which some studies have suggested was appropriate for arch scans [35].

The Trios 5 scanning system has the highest absolute accuracy (absolute average of 0.112 mm) among the evaluated systems. These findings align with those of previous studies [34]. Even though the digital scanners examined in different studies are not identical, there are numerous potential scanning environments and processes, and there is currently no standardized protocol.

## 5. Conclusions

Despite limited comparative studies on the accuracy of the latest generation of intraoral scanners for evaluating full arch impressions, our research fills this gap by conducting a comprehensive comparative study of the latest intraoral scanners available on the market as of December 2023: Trios 5—3SHAPE, Copenhagen, Denmark, CEREC Primescan—Dentsply Sirona, New York, NY, USA, Planmeca Emerald S—Planmeca Oy, Helsinki, Finland and Medit i700—Medit Corp, Seoul, Republic of Korea. Importantly, our study adhered strictly to the ISO standard for assessing the accuracy of digital impression devices.

Our findings underscore a notable disparity between the accuracy requirements of conventional impression materials for dental arch impressions and the significantly higher accuracy levels achieved by all examined digital scanning methods. While some studies have reported similar levels of trueness between digital and conventional systems, our research reveals significant variations in full arch impression accuracy between digital intraoral scanners and conventional impression materials.

Across all evaluated digital intraoral scanners, we observed variations in full arch impression accuracy. Trios 5 emerged as the most accurate in recording data, with Planmeca Emerald demonstrating an exceptionally low overall deviation. Our results also indicate a statistical difference between these scanners and those from previous generations, suggesting advancements in hardware and software have led to more precise results. The most recent hardware and software version of the Medit generated more precise results. Moreover, the output of all four scanners falls within a clinically acceptable range for full arch digital impression utility.

Integrating advanced intraoral scanners can streamline workflows, reduce chairside time, and enhance patient experience through more comfortable and efficient scanning processes [36]. Opting for scanners equipped with the most recent hardware and software versions ensures enhanced precision and compatibility with evolving digital workflows.

Despite the limitations inherent in in vitro investigations, our findings suggest that all analyzed intraoral digital scanners are capable of reliably and consistently capturing a full arch scan in dentate patients [36]. However, further investigation is warranted for fully edentulous arches and full arch implant restorations, as the scanning process presents unique challenges [36] and requires evaluation using distinct ISO standard approaches.

## Figures and Tables

**Table 1:** The values of the parameters of the digital test object.

Parameter	Value (mm)
a-b	32
b-c	19,474
c-d	32
a-d	38
a-c	42
b-d	42

**Table 2:** The values of variations between scan data (measured in micrometers).

Value	Trios 5	CEREC	Emerald S	i700
the largest negative deviation	−2000 µm	−2000 µm	−1999 µm	−2000 µm
the largest positive deviation value	2000 µm	2000 µm	2000 µm	2000 µm
Median	11 µm	15 µm	14 µm	6 µm
Average Mean	22 µm	13 µm	29 µm	6 µm
Mean Absolute Deviation (MAD)	112 µm	127 µm	117 µm	114 µm
Root Mean Square Error (RMSE)	165 µm	179 µm	156 µm	174 µm
Standard Deviation (SD)	164 µm	179 µm	153 µm	174 µm
Variance	27 µm	32 µm	23 µm	30 µm
the average of the deviation values’ positive values	126 µm	129 µm	136 µm	117 µm
average of the deviation values’ negative values	−97 µm	−124 µm	−95 µm	−112 µm
(90–10)/2 (Trueness)	168 µm	192 µm	181 µm	161 µm
10% value of deviation values	−141 µm	−189 µm	−146 µm	−154 µm
90% value of deviation values	195 µm	195 µm	216 µm	168 µm
Tolerance	31.00%	26.79%	26.61%	29.11%

## Data Availability

The original contributions presented in the study are included in the article; further inquiries can be directed to the corresponding author/s.
